# Radiology reporting in rectal cancer using magnetic resonance imaging: Comparison of reporting completeness between different reporting styles and structure

**DOI:** 10.1177/20584601241258675

**Published:** 2024-07-01

**Authors:** Gustav Alvfeldt, Peter Aspelin, Lennart Blomqvist, Nina Sellberg

**Affiliations:** 1 Department of Clinical Science, Intervention and Technology, 27106 Karolinska Institutet, Stockholm, Sweden; 2 Department of Molecular Medicine and Surgery, 27106 Karolinska Institutet, Stockholm, Sweden

**Keywords:** abdomen/GI, MR imaging, computer applications-general, health policy and practice, technical aspects

## Abstract

**Background:**

The radiology report is vital for providing imaging information to guide patient treatment, and template-based reporting can potentially increase the reporting completeness. In 2014, a national reporting template for radiological staging of rectal cancer using magnetic resonance imaging (MRI) was implemented in Sweden.

**Purpose:**

To evaluate the impact of the national reporting template by comparing and analysing differences in content and completeness in MRI reports between 2010 and 2016. Focus was to compare reporting completeness (i) between different reporting years and (ii) between three defined reporting styles.

**Material and Methods:**

493 MRI reports were gathered from 10 hospitals in four healthcare regions in Sweden, comprising 243 reports from 2010 and 250 reports from 2016. Reports were classified into three reporting styles: Expanded structured, Minimised structured, and Unstructured, and analysed using qualitative content analysis based on the national template.

**Results:**

In 2010, all reports adhered to Unstructured reporting. In 2016, 44, 42, and 164 reports were conformant to Expanded structured, Minimised structured, and Unstructured reporting, respectively. A comparison between the years revealed a reporting completeness of 48% for 2010 reports and 72% for 2016 reports. Among the 2016 reporting styles, Unstructured reporting had the largest gap compared to the national template, with completeness at 64% versus 77.5% for Minimised structured reporting and 93% for Expanded structured reporting.

**Conclusion:**

Implementation of template-based reporting according to Expanded structure is key to conform to national decided evidence-based practice for radiological staging of rectal cancer.

## Introduction

Colorectal cancer ranks as the third most prevalent cancer in Sweden, with approximately 2100 annual diagnoses of rectal cancer.^1^ The evolving landscape of anti-cancer treatments highlights the need for personalised cancer management.^2^ A central aspect of this approach is the primary staging and restaging of rectal cancer following neoadjuvant therapy, aimed at optimising patient outcomes.^3,4^ Magnetic Resonance Imaging (MRI) is the primary imaging tool for these tasks, with numerous studies emphasising the significance of key prognostic factors in interpreting and reporting MRI findings.^5–8^

Efficient communication and interpretation of findings are the primary objectives of radiologic reporting. However, the diversity in reporting styles indicates the absence of a universally adopted format.^9^ Reporting styles span from unstructured free-text reports to template-based reports with varying levels of structure and detail.^10–13^ Template-based reporting, characterised by standardised and structured formats, has the potential to enhance staging accuracy.^14–16^ Challenges related to the lack of reporting uniformity and the demand for more standardised and structured alternatives have been debated for over two decades, involving radiologists, pathologists, and clinical professionals.^17–20^

In 2014, a national radiology reporting template for primary rectal cancer staging was introduced in Sweden through the Swedish Colorectal Cancer Registry (SCRCR), in collaboration with the Swedish Society of Medical Radiology (SFMR) and the sub-division Swedish Society of Gastrointestinal and Abdominal Radiology (SFGAR).^21,22^

This study aims to assess adherence to the national radiology reporting template for rectal cancer staging by categorising reporting content into three defined styles based on their level of structure: A) ‘Expanded structured’ reports, B) ‘Minimised structured’ reports, and C) ‘Unstructured’ reports.^13^ The primary focus is to compare reporting completeness (i) across different years and (ii) among the three defined reporting styles.

## Material and methods

### Material

The unit of analysis in this study were rectal cancer primary staging reports, divided into two groups:

1. Radiology reports authored in 2010.
2. Radiology reports authored in 2016.

The reports from 2010 were written 3–4 years before the national reporting template for rectal cancer staging was introduced in 2014, but when knowledge from previous research^2–5,23,24^ was available. At the time of the reports from 2016, the national reporting template had been introduced through a national training workshop program and established clinical practice recommendations and guidelines had been available for about 3–4 years.^6,25^

The reports were collected from 10 hospitals in four healthcare regions in Sweden (Table 1). The study protocol was vetted and approved by the Swedish Ethical Review Authority (Ethics approval number: 2017/695-31/5).

**Table 1.** Statistical overview of the reports at each hospital.

Healthcare region	Hospital	Total reports (2010/2016)	Compliant reports (2010/2016)	Selected reports (2010/2016)
Region A	Hospital 2	77/147	30/38	25/25
	Hospital 3	89/113	56/67	25/25
	Hospital 8	106/101	66/55	25/25
Region B	Hospital 9	126/194	72/115	25/25
	Hospital 5	39/74	18/42	18/25
	Hospital 1	67/247	37/51	25/25
Region C	Hospital 10	94/116	88/66	25/25
	Hospital 4	33/74	31/50	25/25
	Hospital 7	44/76	28/47	25/25
Region D	Hospital 6	55/61	39/36	25/25
Total		730/1203	467/567	243/250

Patient reports were identified via the SCRCR and assorted by hospital and radiology department. The healthcare regions and hospitals were chosen based on the number of rectal cancer patients at each site. Regions and hospitals with a high number of patients reported in SCRCR were asked to contribute with data.

Each radiology department provided de-identified reports from their reporting system, filtered by the first pelvic MRI after diagnosis before treatment.

A total of 1933 reports were obtained, 730 from 2010 and 1203 from 2016. Several exclusion criteria needed to be applied on the given reports (Table 2). After applying the listed exclusion criteria, 467 reports from 2010 and 567 reports from 2016 were found compliant (Table 1).

**Table 2.** Exclusion criteria for MRI reports.

#	Exclusion criteria	Comment
1	Demonstration- and/or multi-disciplinary conference reports	These reports already have a previous ‘original’ staging report
2	Reports written other year than 2016	This could happen since year of diagnoses does not always equal year of exam
3	Reports written by sub-contractors and not the actual hospital radiology department	That is, referrals that were forwarded to, and answered by, another radiology department
4	Reports where the examined body part is something else than the abdominal region (abdomen, lower abdomen, pelvis, rectum)	Combinatory procedures are included if they combine abdominal MRI with some other procedure, for example, liver CT for review of metastases
5	Reports where the modality is something else than a pelvic MRI	For example, if the procedure is an abdominal CT
6	Non-staging exams	
7	Follow-up exams	For example, a staging exam post neoadjuvant treatment
8	Cancelled exams	Such exams typically end up with a final report stating that the exam was cancelled in the observations or findings section
9	Exams without rectal tumour findings	For example, if the reason for referral states a rectal cancer staging but the radiology findings doesn’t concur there is a tumour to stage
10	Exams where the ‘reason for study’ equals ‘status post op’ or ‘relapse’	
11	Other kind of tumour findings	For example, anal/colon cancer and not rectal cancer
12	Duplicates	Some datasets contained duplicates of reports

The datasets were staged using Microsoft Excel version 2016 before imported in QSR International Nvivo 11 for coding and analysis. All the compliant reports were randomised in Microsoft Excel using the RAND-function. 25 randomised reports per year from each hospital were imported into Nvivo.

### Methods

The radiology reports were manually interpreted and coded using qualitative content analysis with a pre-defined coding scheme.^26^ With this deductive content analysis method, the content of the radiology reports was coded to pre-defined categories in the coding scheme with a thematic approach, that is, dividing the content of the reports into shorter units or segments with shared thematic meaning.^27–31^

**Fig. 1.**
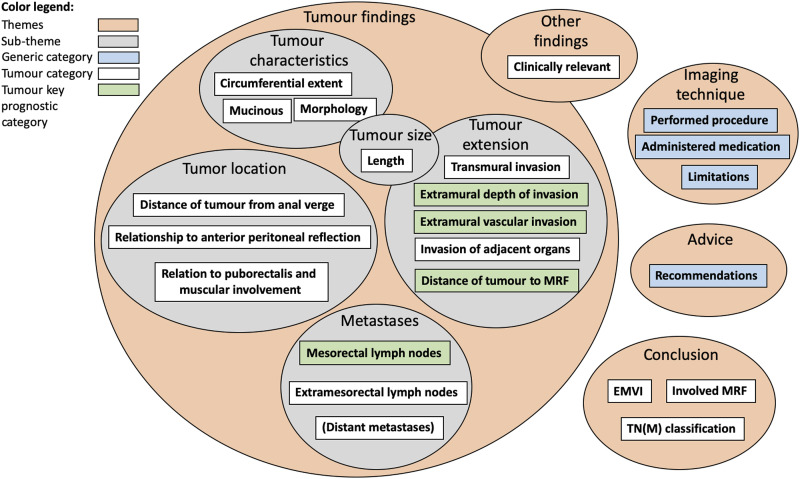
Visualisation of the pre-defined coding scheme.

The pre-defined coding scheme was created consisting of structuring elements^28^ with: (a) themes, (b) sub-themes, and (c) categories (Fig. 1). Categories with close semantical relationship were grouped under a sub-theme within a theme, for example, categories describing the spread of the tumour were grouped to a sub-theme relating to Tumour extension within the theme of Tumour findings.

The coding scheme consist of 18 tumour-specific categories based on the national reporting template for primary rectal cancer staging^21^ and the corresponding SCRCR protocol^32^ with a structure of themes and generic categories conforming to established radiology practice guidelines.^33,34^ 4 of the 18 tumour-specific categories were considered as key categories of prognostic importance.^5^

### Analysis of radiology reports

The specific unit of text to be coded to a category, the recording unit, can be made up of a word, a group of words, a sentence, or an entire paragraph,^29,35^ that is, one sentence can contain many recording units and be semantically divided into shorter units and coded to different categories.

The recording units within the MRI reports were coded to a category, based on its meaning. Recording units were coded to the same theme and category if they shared the same logical semantics.^28,29,36^

**Fig. 2.**
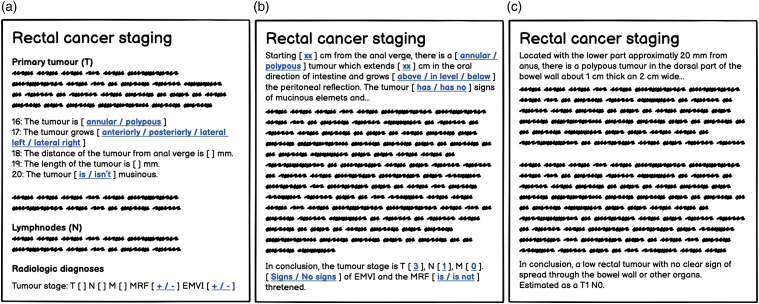
Examples of reporting styles: (a) Expanded structured reports, a structured protocol with default statements in close relation to the national reporting template. (b) Minimised Structured reports, semi-structured reports with Minimised default statements without apparent relation to the national reporting template. (c) Unstructured reports, narrative reports using free-text prose.

During the analysis, each report was classified to a generic reporting style based on its general composition. The reports were divided into three degrees of structure in accordance with Kelsch et al.; Reporting style A, Expanded Structured reports, Reporting style B, Minimised Structured reports, and Reporting style C, Unstructured reports^13^ (Fig. 2).

By applying this approach on the total cohort of reports, all the recording units of the reporting content were allocated to a specific theme and category (Table 3) and each report were classified to one of the three reporting styles with varying degree of structure. This makes it possible to analyse the reporting completeness of the content and to make comparisons between the different hospitals and healthcare regions in relation to the coding scheme for each reporting style.

**Table 3.** Examples of recording units and how they are coded to a category within a theme.

#	Recording unit	Tumour category	Sub-theme	Theme
1	The tumour covers about ¾ of the circumference with the main growth dorsally and to the left	Circumferential extent	Tumour characteristics	Tumour findings
2	[Discontinuity of the muscularis propria] with smaller (1–5 mm) tumour extensions into the mesorectal fat	Extramural depth of invasion (EMD)	Tumour extension	
3	The shortest distance of the tumour to the mesorectal fascia is 3 mm	Distance of tumour to MRF (CRM)	Tumour extension	
4	The tumour is situated in level with and below the anterior peritoneal reflection	Relationship to anterior peritoneal reflection	Tumour location	
5	In conclusion, a low tumour estimated as T3b/T4b, N1	TN(M) classification	-	Conclusion

Content within square brackets are contextual notes written by the authors to facilitate reading of recording units which has been parted from longer sentences.

### Content comparison in reporting

To facilitate visual comparisons of reporting completeness across various reporting years and styles, we employ radar charts. Reporting completeness is measured by the number of findings documented for each tumour category, containing 18 categories in adherence to the national reporting template. In instances where reports reference multiple rectal tumours, the count of findings can surpass 18 per report. To ascertain relative completeness, we factor in the omitted findings, yielding an average percentage.

For visual consistency within radar charts, all reporting styles (A, B, and C) have been extrapolated to 250 reports each, allowing for side-by-side comparisons in a single chart. Consequently, the radar charts present the same graphical pattern for reporting styles A, B, and C, each representing 250 reports, akin to their original configurations. This approach enables a visual analysis of distinctions both within and among reporting years and various reporting styles within a unified radar chart.

### Content analysis terminology in relation to radiology terminology

The radiology report can be divided into different sections containing different types of information. The main body of the report usually consists of short sentences with descriptions of pertinent positive or negative observations about the radiology examination. These observations are called findings^37^ and correspond to the term recording units in content analysis.

To enhance clarity and readability, the terminology will adapt to radiology terminology, replacing ‘recording units’ with ‘findings’, and the mentioning of the national reporting template will be used as an equivalent to the 18 tumour-specific categories found in the pre-defined coding scheme.

Reporting style A and B will be used as examples of template-based reporting but with reporting style A being labelled as ‘Expanded structured’ and reporting style B as ‘Minimised structured’. Reporting style C consists of free-text reports not based on a template; this kind of reporting will be labelled as ‘Unstructured’ (Fig. 2).

### Limitations, clinical relevance, and trustworthiness

The primary challenge in content analysis is ensuring trustworthiness, which encompasses credibility, dependability, confirmability, transferability, and authenticity.^29–31^ To ensure the validity of the coding scheme, the clinical relevance and trustworthiness of the analyses, a panel of abdominal radiology experts reviewed the scheme, a process that was iterated until the panel reached an agreement on its structure.^28^ The initial coding was done by a medical informatician specialised in imaging informatics (GA). To achieve as high an intercoder consistency and accuracy as possible in the coding process, the coding was double checked by an abdominal radiologist (LB), and spot checked by another radiologist (PA). The study’s sample size was deemed sufficient to reach data saturation and information power^38,39^ for drawing valid inferences about rectal cancer staging reports. Although the reports are dated, they offer insights into the slow evolution of radiology practices, particularly in adapting to new reporting procedures. Despite technological and medical advancements, the slow adoption of structured reporting in clinical practice persists.^40^

Due to the risk that reports can contain errors and that descriptions of findings can be ambiguous and difficult to interpret on account of subjective reasoning and underlying meanings,^17,41–45^ a computer-aided approach to content categorisation has not been chosen for this study as it might reproduce any mistakes if they existed. However, recent advances in deep learning using large language models (LLMs) have only scratched the surface of what that technology could be used for within healthcare, medicine, and research. If developed responsibly and used the right way, LLM-based tools could play a huge part in data management and assist in the analyses, structuring, and coding of texts, such as radiology reports, and could possibly be used as a tool to accurately analyse large amounts of texts much faster and more robust than any qualitative human approach can muster.^46,47^

## Results

**Fig. 3.**
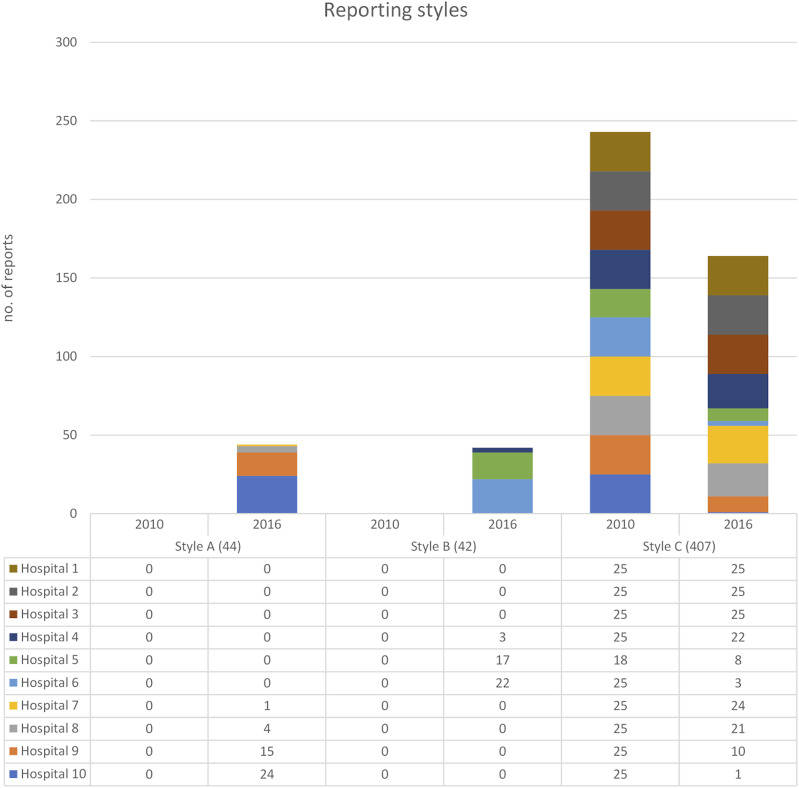
Number of reports per reporting style and year for each hospital.

The 2010 reports were all classified as Unstructured reports (C) with no consistency in structure, number of findings, or standardised terminology. In 2016, 6 out of 10 radiology departments continued to utilise Unstructured reporting (C). Four hospitals adopted template-based reporting at a departmental level, displaying uniform content coverage, structure, and terminology. Two departments implemented Minimised structured reporting (B), using a semi-structured approach with pre-configured prose. Additionally, two departments adopted Expanded structured reporting (A), characterised by a structured protocol closely aligned with the national reporting template (Fig. 3).

Among the six radiology departments still using Unstructured reporting (C) in 2016, two had individual radiologists using Expanded structured reporting (A) and one had individual radiologists using Minimised structured reporting (B). Due to de-identification of data, it remains unclear whether these reports were authored by the same or different radiologists.

### Overall comparison between reporting from 2010 and 2016

**Fig. 4.**
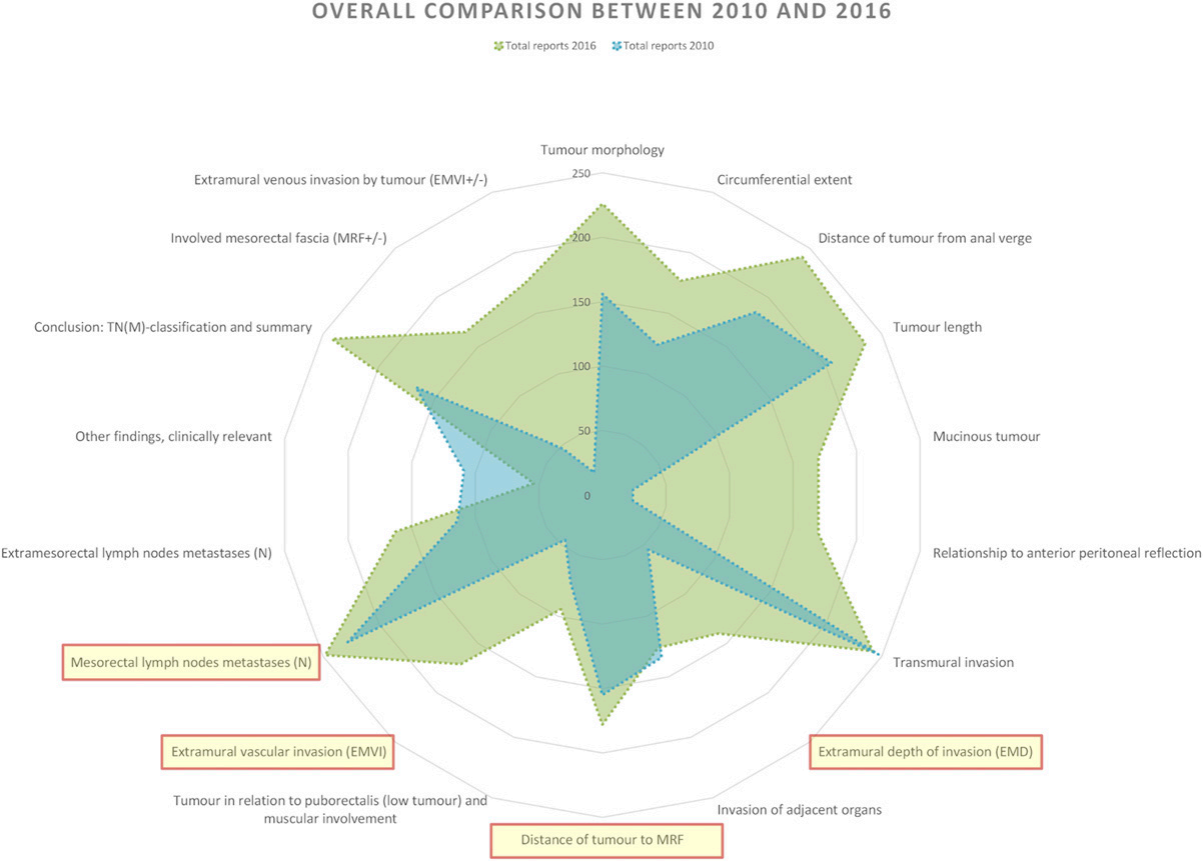
Comparison of findings per tumour category in relation to the national reporting template between all the reports authored in 2010 and all the reports authored in 2016 (no extrapolation). Total information completeness would render a full circle close to the edge of the diagram. Tumour key prognostic categories are marked with light yellow and circled in red.

The result shows differences in reporting practice between reports written in 2010 and 2016. In 2016, reports displayed a more comprehensive representation of findings for nearly all tumour categories. Adjusting for omitted findings, the relative completeness in relation to the national reporting template was 72% in 2016, with an average of 12.9 findings per report out of the 18 tumour-specific categories mentioned. In contrast, 2010 reports scored 48%, with an average of 8.7 findings per report. While the distribution of findings per tumour category follows a consistent pattern across reporting years, the 2016 reports generally report more findings for most categories, although two categories had more findings in the 2010 reports: Invasion of adjacent organs and Other findings (clinically relevant). Some categories showed similar amount of findings in both years, for example, Transmural invasion and Mesorectal lymph nodes metastases (*N*) (Fig. 4).

Notably, when specifically comparing key prognostic categories, there’s an obvious gap between the 2 years in findings reported within the Extramural depth of invasion (EMD) and Extramural vascular invasion (EMVI) categories. The other two categories have similar findings.

Some findings were largely omitted in 2010 reports but more prominently represented in 2016 reports. This pattern also extends to tumour categories within the Conclusion theme.

### Comparison of Unstructured reports from 2010 and Unstructured reports from 2016

**Fig. 5.**
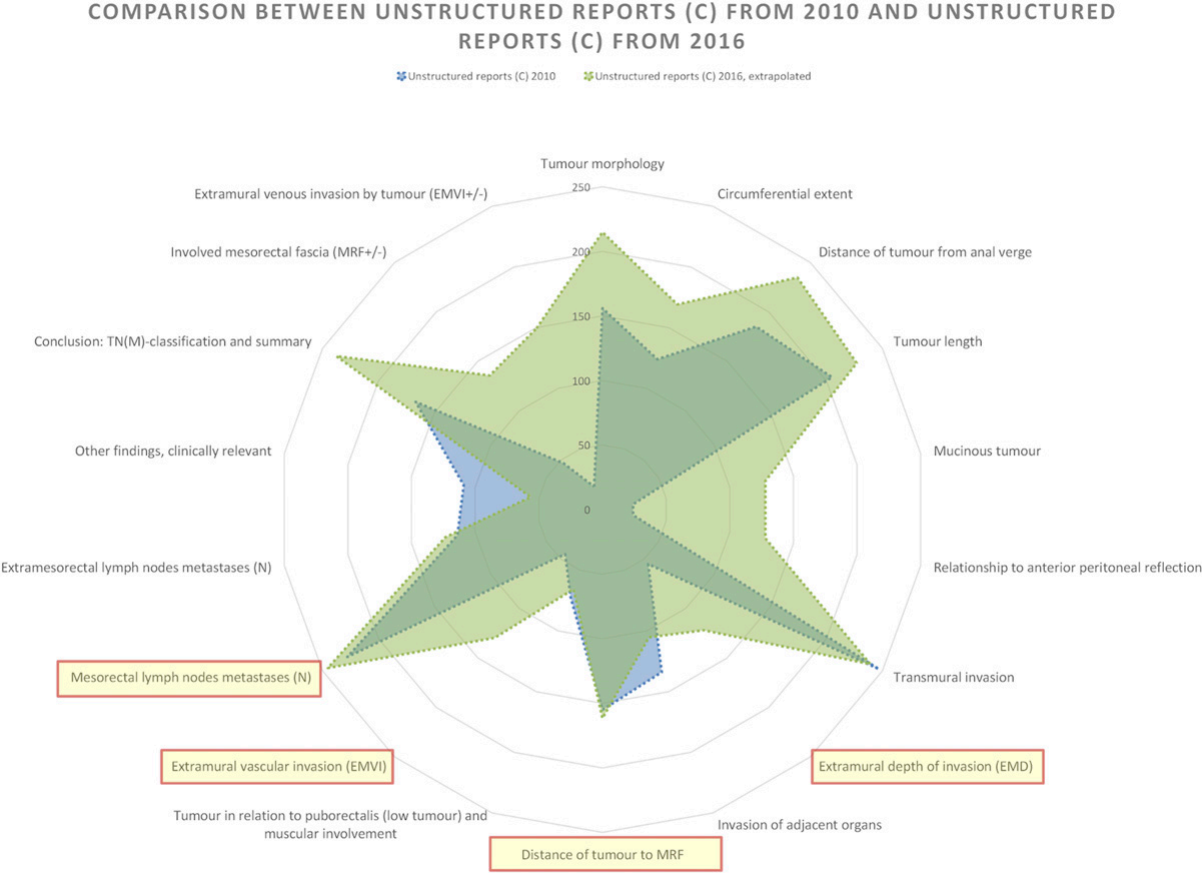
Comparison of findings per tumour category in relation to the national reporting template between all the reports authored in 2010 and the reports authored in 2016 conformant to Unstructured reporting (style C). Total information completeness would render a full circle close to the edge of the diagram. Tumour key prognostic categories are marked with light yellow and circled in red.

When focusing solely on Unstructured reports (style C) between the 2 years, the diagram shapes become more uniform across the reporting years, that is, the distribution of findings per tumour category remains similar between the years but with closer alignment (Fig. 5). Even when only comparing the Unstructured reports, most tumour categories have higher representation in 2016 compared to 2010.

Adjusting for omitted findings, relative completeness for Unstructured reports in 2016 in relation to the national template was 64%, averaging about 11.5 out of 18 findings per report. In contrast, Unstructured reports from 2010 had 48%, averaging 8.7 findings per report.

Notably, the Invasion of adjacent organs category exhibits a more pronounced difference in favour for the 2010 reports (Fig. 5).

The gaps for two of the tumour key prognostic categories are still present for this comparison, but the gaps are smaller. The other two tumour key prognostic categories have higher reporting frequency in the reports from 2016 but not with much difference.

### Comparison of Unstructured reports from 2010 and Minimised structured reports from 2016

**Fig. 6.**
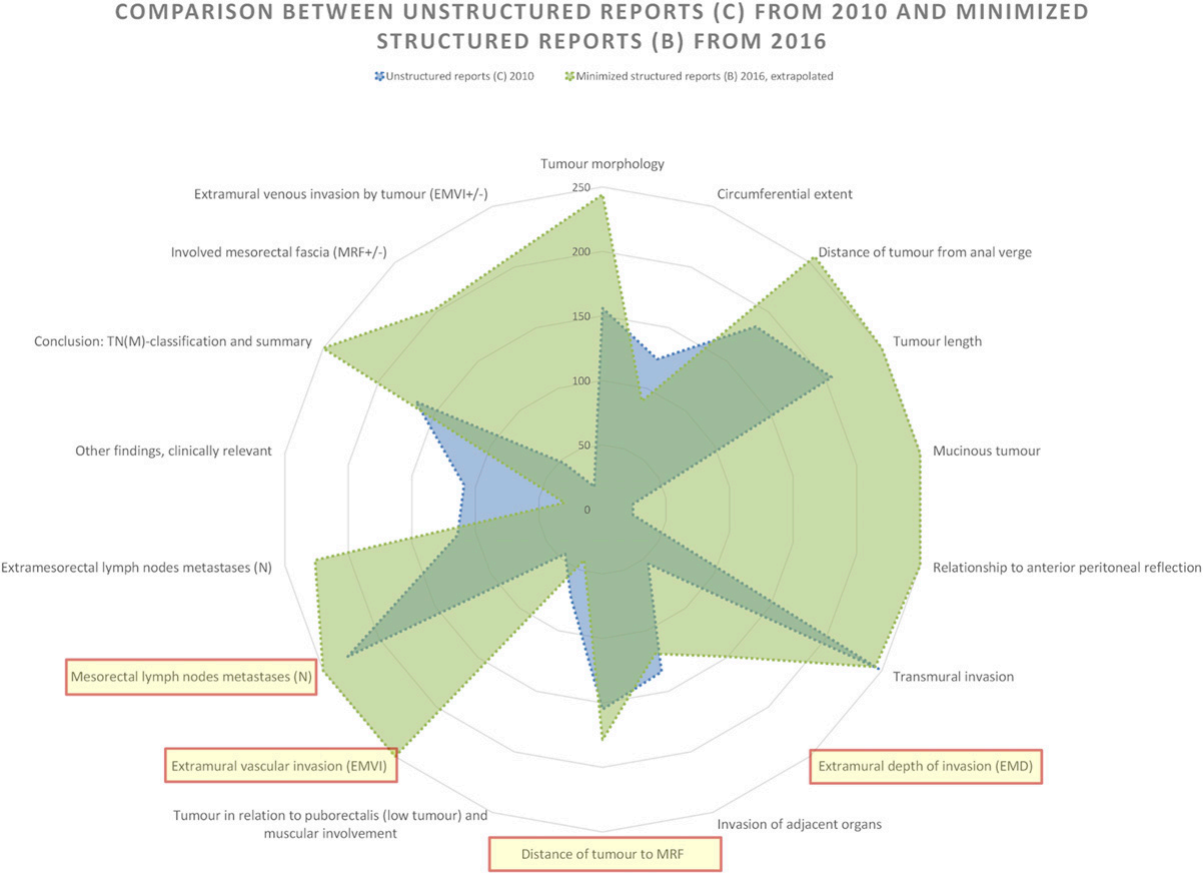
Comparison of findings per tumour category in relation to the national reporting template between all the reports authored in 2010 and the reports authored in 2016 conformant to Minimised Structured reporting (style B). Total information completeness would render a full circle close to the edge of the diagram. Tumour key prognostic categories are marked with light yellow and circled in red.

When comparing 2010’s Unstructured reports to 2016’s Minimised structured reports (Style B), differences become more apparent. The radar chart (Fig. 6) retains similarities, with visible dips indicating low reporting frequencies for similar tumour categories as for the Unstructured reporting. In terms of relative completeness, the Minimised structured reports in 2016 improves to 77.5%, averaging 14 findings per report in comparison to the Unstructured reports from 2010 with its 48% and 8.7 findings per report.

In Minimised structured reporting, it’s notable that many categories with low reporting in 2010 are almost complete in the 2016. At the same time, the number of categories with higher frequency of findings in the Unstructured reporting from 2010 is higher than in any other comparison.

**Fig. 7.**
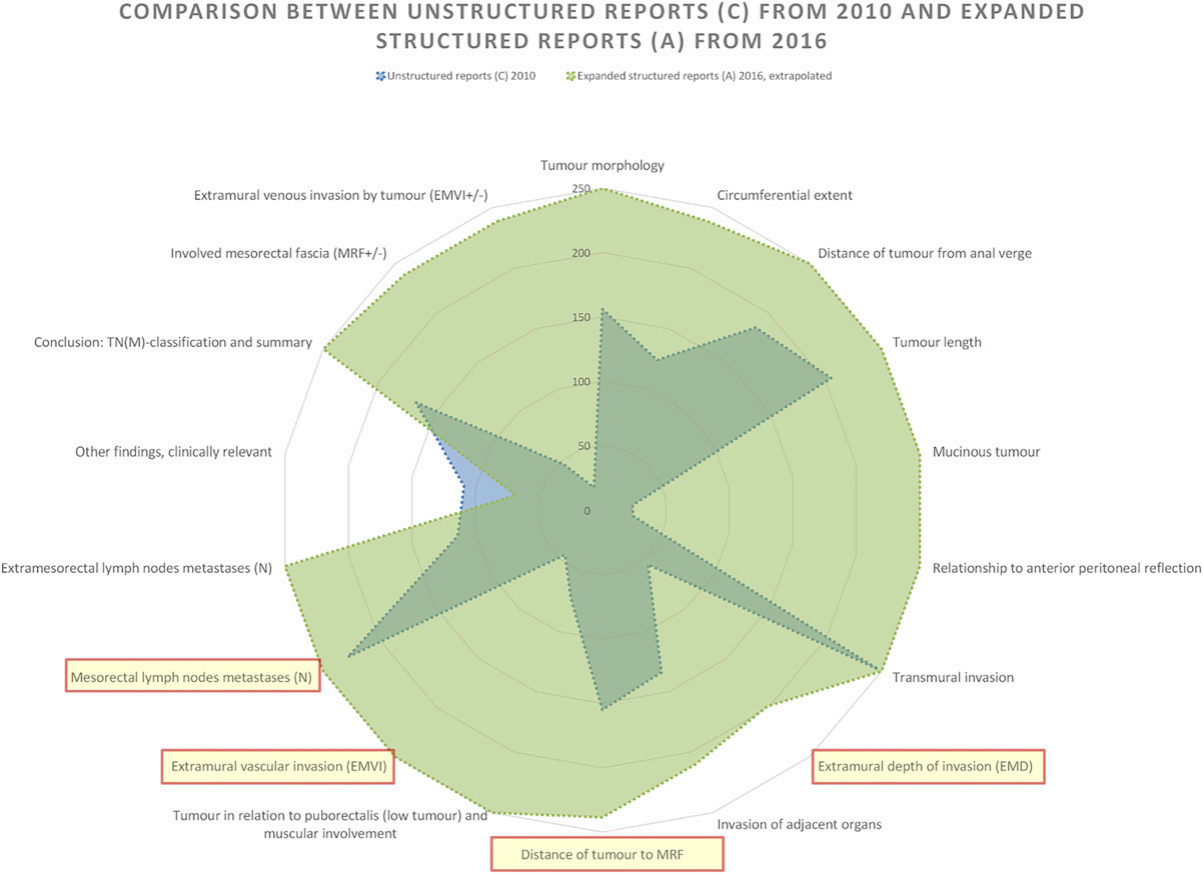
Comparison of findings per tumour category in relation to the national reporting template between all the reports authored in 2010 and the reports authored in 2016 conformant to Expanded structured reporting (A). Total information completeness would render a full circle close to the edge of the diagram. Tumour key prognostic categories are marked with light yellow and circled in red.

Although EMD and Distance of tumour to MRF still have relatively low reporting frequencies, EMVI is highly prioritised in the Minimised structured templates, achieving 100% information completeness (Fig. 9).

### Comparison of Unstructured reports from 2010 to Expanded structured reports from 2016

The biggest difference occurs when comparing the Unstructured reports from 2010 with Expanded structured reports (A) from 2016. The 2016 Expanded structured reports are nearly complete, exhibiting high findings across all tumour categories according to the national reporting template (Fig. 7).

The 2016 Expanded structured reports are nearly complete and highly compliant with the national reporting template, achieving 93% relative completeness with an average of 16.7 findings per report out of 18 possible. This surpasses the 48% for the Unstructured reports from 2010 with its average of 8.7 findings per report by far. The 2016 reports consistently report more findings for each category except one: Other findings (clinically relevant) that have a higher representation of findings in the 2010 reports.

Other than that, the categories of Extramural depth of invasion (EMD), Invasion of adjacent organs, Distance of tumour to MRF, and Circumferential extent have varying degrees of lower coverage but still outperform other reporting styles. However, it is notable that two of these categories are considered as tumour key prognostic categories.

### Comparison between reporting styles within 2016

**Fig. 8.**
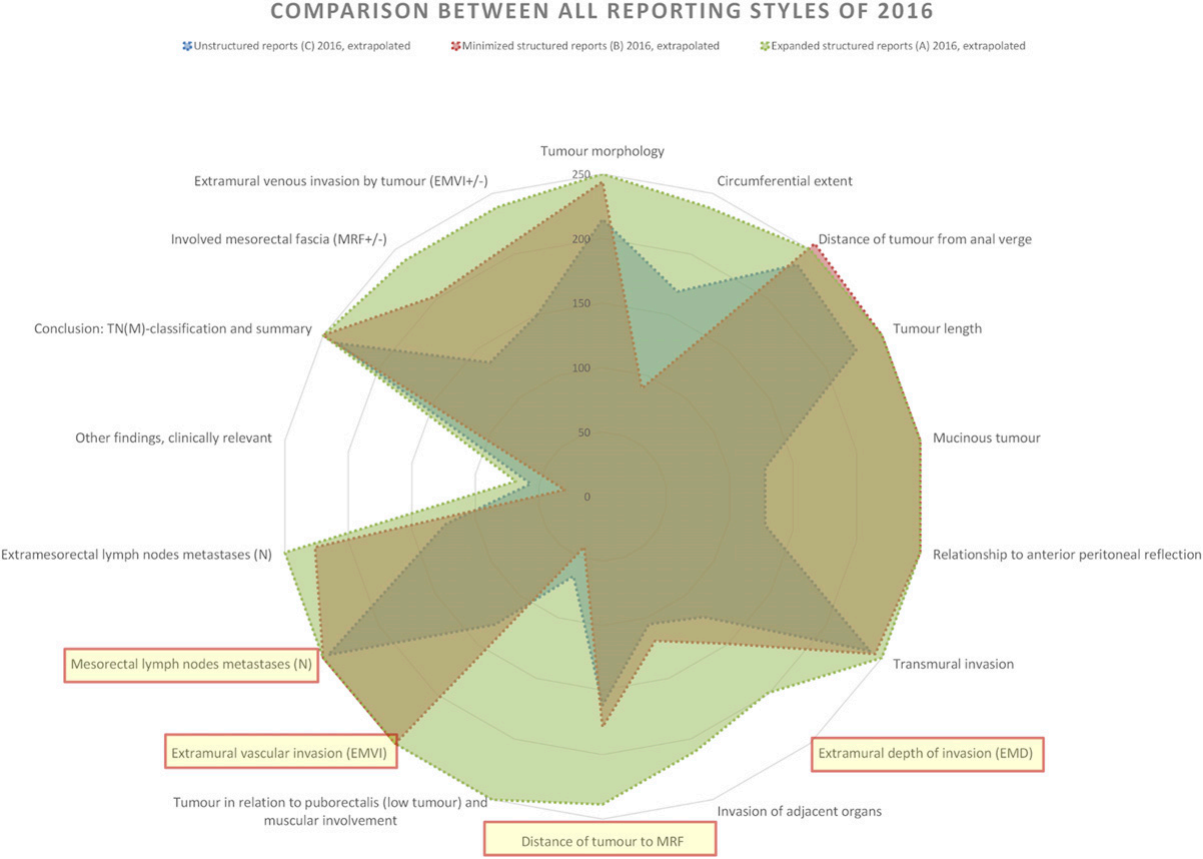
Comparison of findings per tumour category in relation to the national reporting template between all the different reporting styles of 2016. Total information completeness would render a full circle close to the edge of the diagram. Tumour key prognostic categories are marked with light yellow and circled in red.

**Fig. 9.**
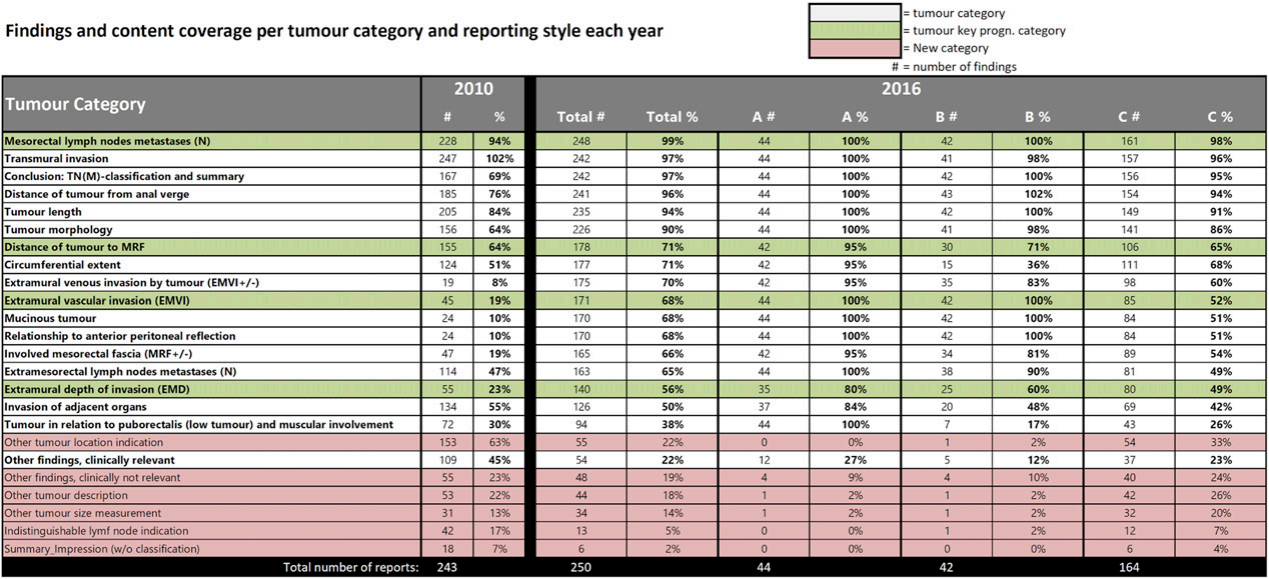
Number of finding and content coverage in percentage per tumour category and reporting year. The recording units from reporting year 2016 has been divided between Expanded structured reports (a), Minimised structured reports (b), and Unstructured reports (c).

In 2016, hospitals using Unstructured reporting (C) showed the greatest discrepancy from the national reporting template. The Unstructured reports covered fewer tumour categories, had fewer findings, and notably fewer categories with peak findings compared to other styles. Minimised structured reporting (B) saw improved information completeness but still had many categories with less than 80% coverage. Compared to Expanded structured reporting (A), more categories had dips in findings frequency, particularly EMD and Distance to MRF, which were omitted nearly as often in Minimised structured reports as in Unstructured reports (Figs. 8 and 9).

Expanded structured reporting (A) demonstrated the highest frequency of findings per tumour category, with all but one category having over 80% coverage, and most reaching 100%. Also, these reports were more stringent with almost no findings mentioned beyond than the ones in the national reporting template. This can also be seen with the Minimised structured reports (B) (Fig. 9).

## Discussion

This study illustrates differences in reporting completeness between rectal cancer staging reports authored in 2010 from those in 2016 in Sweden. Furthermore, it shows variances in structure and completeness based on differences in reporting styles. The reports are categorised into three reporting styles: (A) Expanded structure – structured protocol reports closely aligned with the national reporting template; (B) Minimised structured – semi-structured reports with minimised default statements not directly related to the national reporting template; and (C) Unstructured – free-text prose narrative reports. In 2010, all reports were Unstructured, while those from 2016 span all three styles.

Reporting completeness is assessed by measuring the gap between the knowledge concentration within the national reporting template and everyday reporting practice. This gap is based on the proportion of present findings relative to the tumour categories in the national reporting template.

Overall, the gap between reports and the national template is smaller in 2016 than in 2010, regardless of reporting style. In 2010, an average of 52% of the 18 tumour categories were omitted compared to 28% omitted findings in 2016 reports.

Unstructured reporting in 2016 is more complete compared to Unstructured reports from 2010, though the improvement in reporting completeness is less pronounced. Nevertheless, with an overall information completeness of 64% compared to 48%, it’s evident that common knowledge among gastrointestinal radiologists regarding rectal cancer staging improved from 2010 to 2016.

Using Minimised structured and Expanded structured reports, the information completeness increase to 77.5% and 93%, respectively. This suggests that a reporting process guided by a template generates more complete and higher quality reports than Unstructured free-text reporting. However, there is a risk of skewed and selective reporting when using Minimised structured templates. This becomes evident when comparing the Minimised structured reports to either of the other reporting styles regardless of reporting year. This template-reporting style has several categories of findings with 100% or near 100% but are also the reporting style with most gaps in comparison to the Unstructured reports. Not only is the category of Other findings more predominant in the Unstructured reports, but also the categories of Invasion of adjacent organs, Tumour in relation to puborectalis (low tumour) and Circumferential extent of the tumour. Imaging departments needs to be aware of this problem when implementing template-based reporting and acknowledge the need for objectivity in the process of producing the templates so that the templates used doesn’t originate from just one imperious person.

The increased quality of template-based reporting is most evident when measuring the reporting of key prognostic findings, where template-based reports more frequently include key tumour findings and exhibit higher frequency of classified T- and N-stage. This is most obvious when it comes to the Expanded structured reports where the reported findings per report are nearly complete.

The completeness for template-based Expanded structured reports is also higher when it comes to pertinent negatives, or so called ‘findings not present’ and are moreover demonstrating a harmonisation in structure and presentation, as well as in content regarding language, terminology, and units of measurements.

The only category were the Unstructured reports from 2010 supersedes the Expanded structured reports from 2016 is for the Other finding of clinical relevance. The mention of Other findings are higher overall in the reporting from 2010. Imposing on reporting freedom with added restrictions and rules for content that comes when using a template is something that has been mentioned as concerns from the radiology profession in earlier structured reporting research.^48^ However, this might not be the reason in this case since the national reporting template had a category for this kind of information and the fact that our results shows a more frequent use of Other findings in the Expanded structured reporting style than the other reporting styles of 2016, leaving room for more vague interpretation as to why this is the case. It could just be coincidence regarding tumour cases and the fact that it just happened so there were fewer Other findings to be reported in 2016 compared to 2010. But it could also be a shift in reporting focus and resources spent within health care, leading to more reports just answering the exact clinical question without mentioning other types of secondary findings?

There are however still variations in content, even when conformant to a structured reporting template. Variations in reports conformant to the same template can be seen with differences in comparison between hospitals and within a single hospital using the national reporting template. This is something that need to be addressed when it comes to the implementation of structured reporting. The difference in content completeness when comparing the Expanded structured reports with the Minimised structured reports points in favour to implementation of templates developed in consensus within professional societies and associations rather than templates developed by individuals or by singular imaging departments.

Given the slow evolution in the field of radiology workflow practices, the reporting that were observed and recorded a few years ago might not have become obsolete but instead could have evolved or been further refined from 2016 and until now. We find it plausible that many of the insights gained from this study remain pertinent and can be effectively applied to understand and improve the radiology reporting practices of 2024 and beyond. This underscores the value of the study, providing a foundation for continuous improvement and adaptation in radiological reporting techniques.

In conclusion, Expanded structured reporting, as described in this study, significantly enhances information completeness, terminology consistency, and standardised content compared to other reporting styles. There are most likely always opportunities for improvement in the reporting process, but the implementation of template-based reporting is essential for adhering to evidence-based practice although challenges and differences in implementation need further attention.

## Data Availability

The datasets used and analysed for this study are available from the corresponding author on reasonable request.*
